# Greater motor unit discharge rate during rapid contractions in chronically strength-trained individuals

**DOI:** 10.1152/jn.00017.2024

**Published:** 2024-11-11

**Authors:** Jakob Škarabot, Andrea Casolo, Thomas G. Balshaw, Sumiaki Maeo, Marcel Bahia Lanza, Aleš Holobar, Dario Farina, Jonathan P. Folland, Alessandro Del Vecchio

**Affiliations:** ^1^School of Sport, Exercise and Health Sciences, Loughborough University, Loughborough, United Kingdom; ^2^Department of Biomedical Sciences, University of Padua, Padova, Italy; ^3^Versus Arthritis Centre for Sport, Exercise and Osteoarthritis Research, Loughborough University, Leicestershire, United Kingdom; ^4^Faculty of Sport and Health Sciences, Ritsumeikan University, Kusatsu, Japan; ^5^Department of Physical Therapy and Rehabilitation Science, University of Maryland, Baltimore, Maryland, United States; ^6^Faculty of Electrical Engineering and Computer Science, University of Maribor, Maribor, Slovenia; ^7^Department of Bioengineering, Imperial College London, London, United Kingdom; ^8^Department of Artificial Intelligence in Biomedical Engineering, Friedrich-Alexander University, Erlangen-Nürnberg, Erlangen, Germany

**Keywords:** decomposition, motoneuron, resistance training

## Abstract

Though similar motor unit (MU) discharge properties have been observed during slow sustained contractions between chronically strength-trained (ST) and untrained (UT) individuals, it is currently unknown whether differences between these groups exist for when maximal in vivo MU discharge rate is assessed during rapid, maximal rate of force development (RFD) contractions. Therefore, we compared MU discharge characteristics and RFD during rapid contractions in chronic ST and UT individuals. The investigations were performed in two independent cohorts of chronically ST men, with trained elbow flexors (*experiment 1*, *n* = 13, 6 ± 4 yr of training experience) or knee extensors (*experiment 2*, *n* = 11, 9 ± 4 yr of experience), and compared with those of UT (*n* = 12 and *n* = 10, respectively). ST individuals had greater absolute elbow flexion and knee extension RFD throughout the first 150 ms of rapid contractions compared with UT, but this difference was absent for relative RFD. ST exhibited higher initial MU discharge rate in both biceps brachii (74 [68, 80] vs. 56 [50, 63] pulses per second (pps), *P* < 0.0001) and vastus lateralis (102 [90, 115] vs. 76 [63, 90] pps, *P* = 0.0025) and a greater average number of MU discharges per second in both trained muscles in the early phase of rapid contractions. We provide novel evidence for a higher maximal MU discharge rate in strength-trained individuals. Interestingly, despite the augmented output of the spinal cord, no differences in relative RFD were observed, which suggests either greater maximal force enhancement of ST compared with UT and/or slowing of the intrinsic contractile properties by prolonged strength training.

**NEW & NOTEWORTHY** Chronically strength-trained and untrained individuals show similar motor unit discharge rates during slow sustained contractions, however, potential differences in motor unit discharge rates during rapid contractions remained unclear. Here, we show greater maximal motor unit discharge rates during rapid contractions of chronically strength-trained individuals. However, the augmented spinal cord output of strength-trained individuals did not lead to greater relative maximal rate of force development compared with untrained men.

## Graphical abstract

**Figure:**
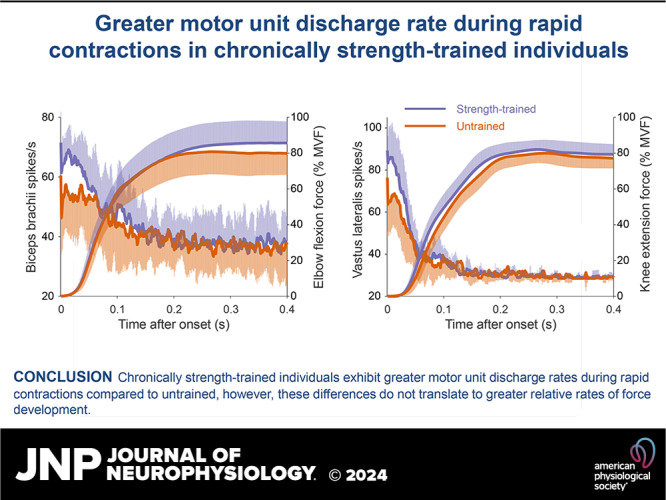

## INTRODUCTION

Strength training results in a significant increase in maximal muscle strength, which, at least within the first weeks of commencing training, is typically accompanied by rapid changes in the neural drive to the muscle (1). Indeed, concomitantly with increased strength, 4 wk of isometric strength training has been shown to increase the discharge rate of a relatively large population of motor units (MUs) (2). Though chronic increases in strength are typically underpinned by superior muscle morphology (3, 4), cross-sectional studies of chronically strength-trained (ST) individuals demonstrate differences in motoneuron excitability (5, 6), reticulospinal excitability (7), and intracortical inhibition (7, 8) and facilitation (7), compared with untrained (UT) controls suggesting that neural adaptations might contribute to the substantially greater strength of chronically trained individuals (9).

Despite some evidence of altered cortical and subcortical input in chronically strength-trained individuals, recent studies suggest no differences in motor unit discharge properties in chronically strength-trained versus untrained individuals (10, 11). However, these studies recorded motor unit activity during slow, sustained (>1 s) submaximal and maximal isometric contractions. In both of these tasks, the motor unit discharge rate is likely to be lower, and thus submaximal, compared with rapid (explosive) contractions that involve increasing force as quickly as possible to maximize the rate of force development (RFD). Indeed, motor unit discharge rates are typically approximately two- to fivefold greater during brief rapid compared with slow sustained contractions (12, 13), and are thus thought to represent the maximal in vivo motoneuron output during isometric tasks (13). Consequently, any neural differences in chronically strength-trained and untrained individuals might be most apparent in conditions where neural output is maximized, such as during rapid contractions. However, when comparing surface electromyography (EMG) amplitude during the rising phase of rapid contraction in strength-trained and untrained individuals, the findings are mixed; studies have found either greater (14) or similar (15) signal amplitude between groups. Importantly, surface EMG amplitude is only a crude indicator of neural drive (16) and thus a more sensitive measure of neural drive may be required to ascertain whether chronically trained individuals exhibit alterations in maximal in vivo motoneuron output.

Investigations of the performance of rapid contractions in strength-trained compared with untrained individuals have consistently found greater absolute rate of force development (14, 15). However, relative rate of force development, which assesses the ability to express the available force-generating capacity in a rapid situation has produced more mixed findings. For example, strength-trained individuals have been found to exhibit greater (14), similar (17), or smaller (15) relative rate of force development compared with untrained individuals. In addition, studies have also found slower intrinsic contractile properties (e.g., relative rate of force development and time-to-peak-tension during evoked contractions) in strength-trained individuals (15) that could counteract any differences in neural drive.

In this study, we assessed the rate of force development and motor unit discharge characteristics of two different groups of chronically strength-trained individuals, with trained elbow flexors or knee extensors, respectively, and compared them to untrained controls during the performance of rapid contractions. We hypothesized that strength-trained individuals would exhibit a greater initial motor unit discharge rate but the difference in motor unit discharge rate between groups would be of insufficient magnitude to lead to differences in the relative rate of force development.

## MATERIALS AND METHODS

### Participants

Two separate cohorts were involved in this study as part of investigations into chronically strength-trained elbow flexors [*experiment 1* (10)] and knee extensors [*experiment 2* (11)]. The experiments were approximately two years apart and no participants took part in both experiments. Experimental procedures were approved by the Loughborough University Ethical Committee (R17-P174 and 2021-1749-3524) in accordance with Declaration of Helsinki. For both experiments, the general inclusion criteria included age (18–40 yr), and lack of current neuromuscular or musculoskeletal conditions affecting the function of joints involved in the study. Participants were excluded if reporting the use of anabolic or androgenic pharmacological agents. To be considered strength-trained, participants had to report at least 3 yr of consistent strength training experience (>10 mo a year), of which at least 2 or 1 session per week had to be devoted to upper arm (for *experiment 1*) and leg training (for *experiment 2*), respectively. In addition, individuals in the strength-trained group had to exceed 90 Nm (*experiment 1*) and 800 N (*experiment 2*) of isometric torque and force, respectively. To be considered untrained, individuals had to report no history of strength training or partaken in systematic training of any type in the past 18 mo.

For the elbow flexor experiment, 13 strength-trained (means ± SD, age: 24 ± 4 yr, stature: 1.81 ± 0.09 m, mass: 88.6 ± 14.3 kg, IPAQ: 5,453 ± 2,825 metabolic equivalent·min·wk^−1^; 6 ± 4 yr of strength training experience) and 12 untrained (21 ± 2 yr, 1.78 ± 0.07 m, 75.7 ± 11.2 kg, 2,787 ± 1,471 metabolic equivalent·min·wk^−1^) men were included. Strength-trained individuals were of similar height (Student’s *t* test; *P* = 0.3473), but were older (*P* = 0.0244), heavier (*P* = 0.0194), and more physically active (*P* = 0.0077) compared with untrained in the elbow flexor experiment. On average, strength-trained individuals reported the use of near-maximal (1–5 repetition maximum, RM), heavy (6–14 RM), and moderate (>15 RM) loads for 38 ± 28, 49 ± 27, and 13 ± 11% of their training time, respectively, and 11 of 13 participants reported performing training by moving loads as fast as possible; of these 13 participants, seven reported doing so regularly (1–3 times per week), four did this training occasionally (<1 per wk), and two did not perform this type of training. In the knee extensor experiment, 11 strength-trained (25 ± 2 yr, 1.78 ± 0.04 m, 85.8 ± 7.0 kg, 4,898 ± 2,617 metabolic equivalent·min·wk^−1^; 9 ± 4 yr of strength training experience) and 10 untrained (24 ± 2 yr, 1.80 ± 0.08 m, 74.2 ± 8.4 kg, 2,659 ± 1,555 metabolic equivalent·min·wk^−1^) men participated. Compared with untrained, strength-trained individuals were heavier (*P* = 0.0030) and more physically active (*P* = 0.0280), but of similar height (*P* = 0.5685) and age (*P* = 0.4638) in the knee extensor experiment. On average, strength-trained individuals reportedly spent 37 ± 23%, 49 ± 21%, and 14 ± 21% of their training time using near-maximal, heavy, and moderate loads, respectively, with 7 of 11 participants reported occasionally having performed training by moving loads as fast as possible, of which two participants reported doing so regularly (1–3 times per wk). In the interest of transparency, the sample size in both experiments was smaller than the previously published reports from these larger investigations (10, 11); this is due to the inability to reliably identify motor unit discharges in some individuals.

### Experimental Design and Protocol

Participants attended the laboratory twice, once for familiarization where they practiced performance of rapid voluntary isometric contractions, and for an experimental session. Contractions were performed unilaterally, with the nondominant limb in *experiment 1* (elbow flexors), whereas they were performed with the dominant limb in *experiment 2* (knee extensors). Previous research has shown that limb dominance had no effect on maximal isometric strength and motor unit discharge characteristics (18). The visits were separated by at least two, but no more than seven days. Participants were instructed to avoid strenuous activity for two days prior, and caffeine on the day of the experimental session.

**Figure 1.**
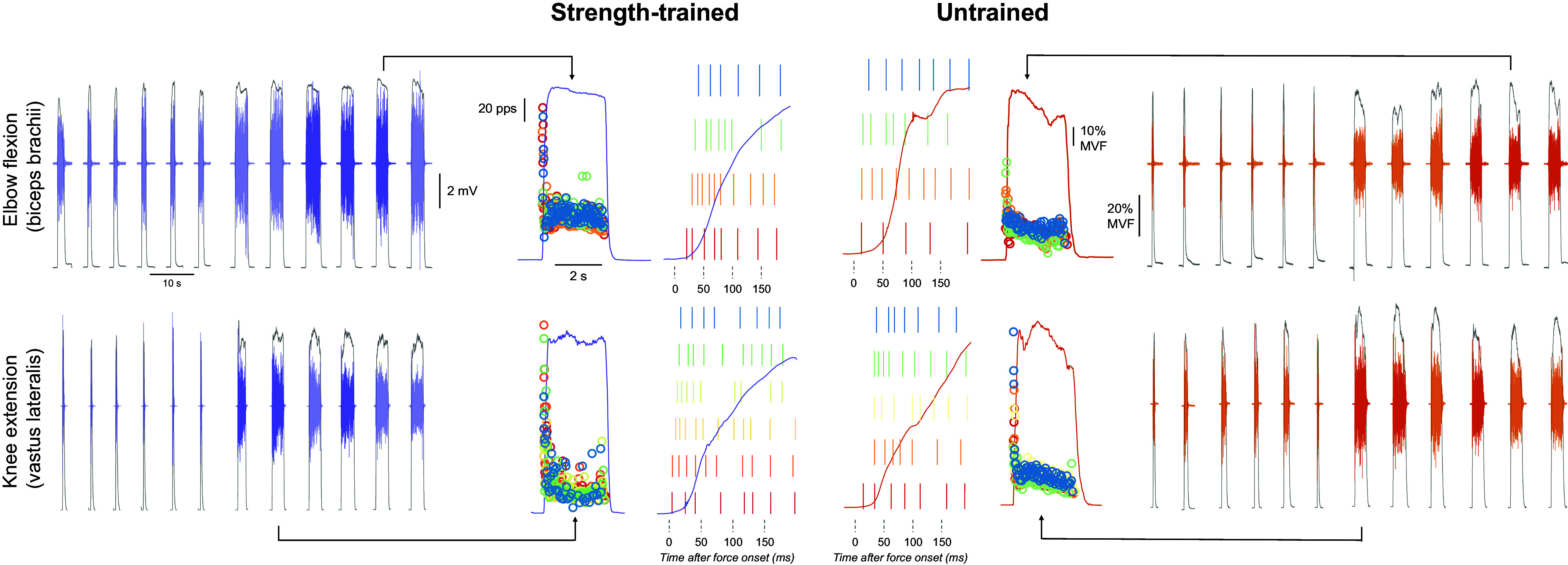
Data acquisition and analysis. Example elbow flexion (*top row*) and knee extension (*bottom row*) force traces of two individuals from the strength-trained (purple) and untrained (orange) groups during the performance of six rapid and six rapid-hold contractions to at least 80% of maximal voluntary force (MVF), along with the electromyographic activity of biceps brachii and vastus lateralis, respectively (n.b. only 1 of 64 channels is shown for clarity). The electromyographic (EMG) signals from rapid-hold contractions were decomposed into individual motor unit spike trains, from which discharge rate was calculated; examples of contractions are displayed with instantaneous discharge rates (in pulses per second, pps) presented in circles. The rate of force development was calculated in the first 150 ms from force onset for the best three rapid-hold contractions (based on force at 100 ms; denoted by the bolder color of EMG) as seen in example traces in the center that showcases the force signal from −20 to 200 ms relative to force onset along with a raster plot of individual motor unit discharges.

Following skin preparation and electrode placement, participants were seated on a custom-made isometric dynamometer. They were initially instructed to perform a series of submaximal unilateral isometric voluntary contractions of ∼3–5 s in duration (3 × 50%, 3 × 75%, and 1 × 90% of perceived maximal voluntary force) for the purposes of warm-up. Following warm-up, maximal isometric efforts of 3–4 s duration were performed by participants to assess maximal voluntary force (MVF). Two trials were performed separated by 60 s of rest, and an additional trial was performed if the first two differed by more than 5%. The highest instantaneous force value achieved during these trials was taken as MVF. After determining MVF, participants were tasked to perform six rapid contractions (separated by 30 s) whereby they were instructed to start from rest and then produce force “as fast and as hard as possible” reaching at least 80% MVF as quickly as possible (Fig. 1). Contractions were considered valid if participants did not exhibit countermovement or pre-tension (>0.5 N). Trials that did not meet the validity criteria were discarded and repeated. Following this block of six rapid contractions and another 3–5 min of rest, participants performed six rapid-hold contractions, separated by ∼30 s of rest (Fig. 1). Though previous studies have used as many as 12 contractions when assessing the rate of force development, we display similar intraindividual variability of rapid-hold contraction performance between the groups in both experiments (Supplemental Table S1; see https://doi.org/10.6084/m9.figshare.26789488), suggesting the use of fewer trials is an unlikely factor in group comparisons. The instructions for the performance of rapid-hold contractions were the same as for rapid ones, but participants were additionally instructed to maintain the 80% force target for ∼2–3 s. The increased contraction duration allows for a greater number of motor unit action potentials to be recorded, which is necessary for the high-density surface electromyography (HDsEMG) signal decomposition during rapid contractions (13). In addition to displaying live force-time feedback on a monitor ≈1 m away (*y*-axis set to −5% to 100% MVF; 30-s resolution) with a horizontal cursor denoting the 80% MVF minimal target, participants also received live feedback on the rate of force development (50 ms slope of the force-time curve), with their best performance highlighted by a horizontal cursor corresponding to the peak slope that served as a target for subsequent contractions (19). Displaying this performance feedback ensured that participants maintained the performance of rapid-hold compared with rapid contractions. Indeed, we have previously shown that with the appropriate instruction and instant display of performance (force-time, rate of force development) the addition of a ∼3-s plateau had no influence on the performance of rapid contractions (20).

### Experimental Procedures

#### Force recording.

Elbow flexor force recordings were performed with participants seated upright (90° hip angle) in a rigid custom-made isometric dynamometer, the shoulder of the nondominant arm flexed at 90° with a slight horizontal abduction (10°), the posterior of the upper arm resting on a rigid horizontal board with the elbow flexed at 70° (0° = full extension) and forearm half-supinated at 45°. The wrist of the nondominant arm was strapped to an adjustable brace that was connected perpendicular and in-line with a calibrated S-beam strain gauge (Force Logic, Swallowfield, UK).

Knee extensor forces were recorded with participants seated in a rigid custom-made isometric dynamometer equipped with a calibrated S-beam strain gauge (Force Logic, Swallowfield, UK), with the knee and hip flexed at 65° and 54°, respectively (0° = full extension). To prevent extraneous movement, participants were tightly strapped across the chest and pelvis. The dominant leg was strapped at ∼15% of tibial length, defined as the distance between lateral malleolus to the center of the knee joint, above the ankle and in-series with a calibrated strain gauge (described earlier). The configuration used in the knee extension experiment has been used previously by our group and has been shown to maximize knee extensor forces (21).

The joint angle configuration for both studies was chosen according to previous studies from our group (15, 22–24) that provided reference values for the strength-trained group (see *Participants* for details). The analog force signal was amplified (200× for elbow flexors, 370× for knee extensors), and sampled at 2,048 Hz (16-bit multichannel amplifier, Quattrocento; OT Bioelettronica, Torino, Italy). To display live force feedback (monitor placed ∼1 m in front of participants), the analog force signal was also simultaneously sampled with an analog-to-digital converter (2,000 Hz, Micro 1401-3 and Spike2 v.10 software, CED Ltd., Cambridge, UK).

#### High-density electromyography.

High-density EMG signals were recorded by placing two grid arrays of 64 equally spaced electrodes on the agonist muscles of interest. Before electrode placement, the area was shaved, and the skin lightly abraded and cleansed with alcohol. For the elbow flexors, the array electrodes (64 channels, 13 rows × 5 columns, 1 mm electrode diameter, 8 mm interelectrode distance; ELSCH064NM2, OT Bioelettronica) were placed on short and long heads of biceps brachii identified through palpation by an experienced investigator who outlined the profile of both muscles with a surgical marker. The center of the array electrode was positioned over the proximal-distal center of the muscle belly. The reference and the main ground electrode (dampened strap) were placed over the radial styloid process and ulna styloid process, respectively. For the knee extensors, electrode arrays (64 channels, 13 rows × 5 columns, 1 mm electrode diameter, 8 mm interelectrode distance; GR08MM1305; OT Bioelettronica) were placed on the vastus lateralis and medialis muscle bellies as described previously (25). The reference electrodes (Kendall Medi-Trace, Canada) were placed over the patella, and a dampened strap placed on the ankle served as the main ground electrode. The longitudinal axes of the bi-dimensional arrays were also aligned with the assumed anatomical direction of fibers for both elbow flexors and knee extensors. The placement of electrode arrays was facilitated by disposable bi-adhesive foam layers (SpesMedica, Battipaglia, Italy), which have the holes corresponding to the electrodes on array that we filled with conductive paste (AC Cream, SpesMedica). All HDsEMG signals were recorded in monopolar derivation, band-pass filtered (10–500 Hz), sampled at 2,048 Hz, and digitized using a 16-bit amplifier (Quattrocento; OT Bioelettronica).

### Data Analysis

#### Force signal.

The voltage force signal was first converted to force (N) and gravity was corrected. Initially, a zero-lag low-pass Butterworth filter with a 400 Hz cut-off frequency was applied to the signal. The onset of force was then visually determined by a trained investigator using a systematic approach (26). After the force onset was determined, additional filtering of the signal with zero-lag low-pass Butterworth filter with a 20-Hz cut-off frequency was applied. The rate of force development (N·s^−1^), defined as the first derivative of the force signal, was then calculated in the fixed time intervals following force onset, 0–50 ms, 0–100 ms, 0–150 ms, 50–100 ms, and 100–150 ms to allow for comparison with other studies (15) and because different neuromuscular factors influence the rate of force development in the sequential time periods (19, 27). The maximal rate of force development was calculated as the maximal value of the first derivative of force calculated for overlapping time windows from 0–1 up to 0–200 ms (RFD0-Xmax, N·s^−1^). The best three trials based on the force produced at 100 ms following force onset and that displayed no countermovement or pre-tension (<0.5 N), and with sufficiently high force output (>80% MVF), were selected for final analysis.

#### High-density electromyography signal.

Monopolar HDsEMG signals from the rapid-hold contractions were initially band-pass filtered (fifth-order, zero-lag Butterworth; 20–500 Hz). Channels that exhibited poor signal-to-noise ratio, poor skin-electrode contacts, and movement artifacts were removed using a semiautomated custom-made Matlab tool based on area under the power spectrum and amplitude. HDsEMG signals were then decomposed into motor unit spike trains using the Convolution Kernel Compensation algorithm (28). To increase the number of firings and thus improve decomposition accuracy (29), all six rapid-hold contractions were concatenated before decomposition. Motor unit spike trains were subjected to editing using established approaches by an expert operator (30), with only motor units exhibiting a reliable discharge pattern and a pulse-to-noise ratio ≥30 dB retained (29). Only data from the biceps brachii short head for elbow flexors, and vastus lateralis for knee extensors were analyzed because of better signal quality, and because they yielded reliable and accurate identification of motor units in a significantly greater proportion of individuals.

From decomposed and processed high-density EMG signals, we calculated the discharge rate (Fig. 1) at initial firing (the average of the reciprocal of the first three interspike intervals) and at the plateau of the rapid-hold contractions (the average of the reciprocal of the 10 interspike intervals at 300 ms from contraction onset). Furthermore, we calculated the coefficient of variation of the interspike interval at the plateau of rapid-hold contractions and the cumulative discharge rate from the first discharge to 400 ms. The average discharge rate per motor unit per second (i.e., normalized cumulative discharge rate) was then calculated by a moving 35 ms epoch from the first motor unit discharge, shifted every 1 ms for 400 ms, and was normalized to the time epoch and the number of active motor units in a given epoch (13). Only the contractions corresponding to the best three trials based on force output (see *Force signal*) were included in the final analysis.

### Statistical Analysis

All statistical analyses were performed in R studio (v.1.4.1106, R Foundation for Statistical Computing, Vienna, Austria). Shapiro–Wilk test and quantile-quantile plots were used to confirm normal distribution of residuals. Differences between groups in the number of identified motor units were assessed with Student’s *t* test. Differences between groups in maximal isometric strength were assessed using a linear model. Between-group differences in the rate of force development, motor unit discharge rate during the initial and plateau period of the contraction and coefficient of variation of the interspike interval on the plateau of the contraction were assessed using separate linear mixed-effect models (*lme4* package; 31) with group (strength-trained, untrained) as fixed effect and participant as random intercept. To assess between-group differences in the average discharge rate per motor unit per second an additional fixed factor of time (sequential 35-ms time windows as this period is equivalent to a typical neuromechanical delay; 13) was added to the model. Note that the best three trials for each individual were used in the model. Analysis of variance (Type II Wald χ^2^ test) was performed to assess the statistical significance of the linear effects model (*lmerTest* package; 32). Estimated marginal means with a 95% confidence interval (Tukey’s correction) and effect sizes (Cohen’s *d*; the difference between estimated marginal means divided by the residual standard deviation of the linear mixed model) were calculated to present the differences between groups (*emmeans* package; 33). A linear regression was performed to assess the association between maximal rate of force development and motor unit discharge rate. Significance was set at an α level of 0.05. Unless stated otherwise, data are presented as estimated marginal means [95% confidence interval].

## RESULTS

A total of 74 motor units were identified following decomposition of biceps brachii HDsEMG signals, of which 42 were identified in strength-trained (means ± SD per contraction per individual; 3.2 ± 1.8, range: 1–6) and 32 in untrained individuals (2.7 ± 1.9, range: 1–7; Supplemental Table S2; see https://doi.org/10.6084/m9.figshare.26789488), with no differences between groups (*P* = 0.4500). In vastus lateralis, 74 motor units were identified in total; 43 were identified in strength-trained (3.9 ± 2.2, range: 2–8) and 31 in untrained (3.1 ± 1.7, range: 1–6; Supplemental Table S2; see https://doi.org/10.6084/m9.figshare.26789488). The number of identified motor units in vastus lateralis did not differ between strength-trained and untrained (*P* = 0.3531). Examples of motor unit discharges identified during rapid contractions in a strength-trained and untrained individual during elbow flexion and knee extension are shown in Fig. 1.

**Figure 2.**
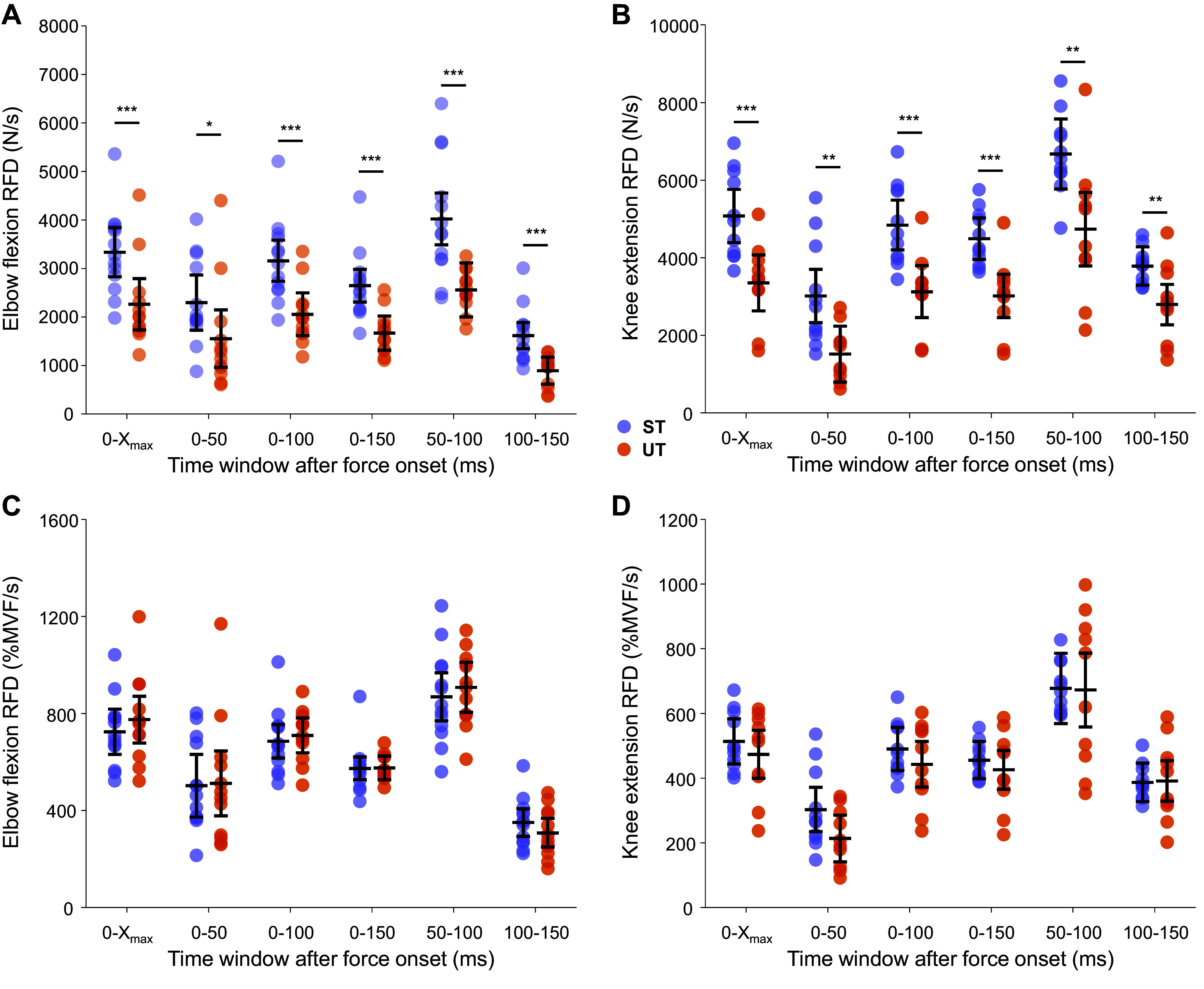
Elbow flexor (*A* and *C*) and knee extensor (*B* and *D*) rate of force development in strength-trained and untrained individuals. The absolute (*A* and *B*) and relative (expressed as a proportion of maximal voluntary force, MVF; *C* and *D*) rate of force development (RFD) in the 0-X_max_, 0–50, 50–100, 100–150, 0–100, and 0–150 ms time windows following force onset during rapid isometric elbow flexion (*A* and *B*) and knee extension (*C* and *D*) in strength-trained (ST, purple; elbow flexion: *n* = 13, knee extension: *n* = 11) and untrained (UT, orange; elbow flexion: *n* = 12, knee extension: *n* = 10) individuals. The lines with error bars represent the estimated marginal means with 95% confidence intervals, with filled circles denoting individual participant averages across three best contractions (based on force achieved in the first 100 ms). ***P < 0.001, **P < 0.010, *P < 0.50 relative to the other group.

Strength-trained individuals were on average 59% and 39% stronger during maximal isometric elbow flexion (457 [424, 490] vs. 287 [253, 321] N, *d* = 2.96; *F*(1) = 54.8, *P* < 0.0001) and knee extension (985 [920, 1,049] vs. 709 [642, 776] N, *d* = 2.71; *F*(1) = 38.6, *P* < 0.0001), respectively. With both the elbow flexors and knee extensors, strength-trained individuals produced greater absolute forces and maximal rate of force development (elbow flexion: 3,337 [2,829, 3,845] vs. 2,265 [1,736, 2,794] N·s^−1^, *d* = 3.67; χ^2^(1) = 9.1, *P* = 0.0025; knee extension: 5,081 [4,394, 5,768] vs. 3,357 [2,636, 4,077] N·s^−1^, *d* = 4.14; χ^2^(1) = 13.1, *P* = 0.0003), but no differences were noted when the maximal rate of force development was normalized to maximal isometric strength (elbow flexion: χ^2^(1) = 0.6, *P* = 0.4446; knee extension: χ^2^(1) = 0.7, *P* = 0.4122, respectively; Fig. 2).

Strength-trained individuals also produced greater rates of force development in specific time windows after force onset (Fig. 2, *A* and *B* for elbow flexion and knee extension, respectively), but these differences were not evident when rates of force development were normalized to maximal isometric strength (Fig. 2, *C* and *D*, respectively). Specifically, strength-trained individuals produced greater absolute rates of elbow flexor force development compared with untrained in the 0–50 (2,298 [1,727, 2,870] vs. 1,554 [960, 2,149] N·s^−1^; *d* = 1.60; χ^2^(1) = 6.2, *P* = 0.0130), 0–100 (3,161 [2,735, 3,586] vs. 2,057 [1,614, 2,499] N·s^−1^; *d* = 6.12; χ^2^(1) = 13.8, *P* = 0.0002), 0–150 (2,647 [2,308, 2985] vs. 1,670 [1,317, 2,022] N·s^−1^; *d* = 11.3; χ^2^(1) = 17.1, *P* < 0.0001), 50–100 (4,023 [3,488, 4,559] vs. 2,559 [2,002, 3,116] N·s^−1^; *d* = 3.67; χ^2^(1) = 15.3, *P* < 0.0001), and 100–150 ms (1618 [1,348, 1,888] vs. 895 [615, 1,176] N·s; *d* = 3.98; χ^2^(1) = 14.7, *P* = 0.0001) time windows after force onset. Similar to the results for elbow flexion, absolute knee extensor rate of force development was greater in strength-trained individuals in the 0–50 (3,019 [2,330, 3,707] vs. 1,522 [800, 2,244] N·s^−1^; *d* = 1.76; χ^2^(1) = 9.9, *P* = 0.0017), 0–100 (4,848 [4,206, 5,490] vs. 3,132 [2,459, 3,805] N·s^−1^; *d* = 4.52; χ^2^(1) = 14.9, *P* = 0.0001), 0–150 (4,495 [3,961, 5,030] vs. 3,021 [2,461, 3,582] N·s^−1^; *d* = 5.94; χ^2^(1) = 15.9, *P* < 0.0001), 50–100 (6,678 [5,775, 7,580] vs. 4,742 [3,795, 5,689] N·s^−1^; *d* = 3.67; χ^2^(1) = 9.6, *P* = 0.0020), and 100–150 ms (3,790 [3,293, 4,287] vs. 2,799 [2,277, 3,320] N·s^−1^; *d* = 3.25; χ^2^(1) = 8.3, *P* = 0.0040) time windows after force onset. Conversely, no differences in the relative rate of force development were noted between the groups for any time window for both elbow flexors (0–50 ms: χ^2^(1) = 0.1, *P* = 0.9154; 0–100 ms: χ^2^(1) = 0.3, *P* = 0.6124; 0–150 ms: χ^2^(1) = 0.01, *P* = 0.9498; 50–100 ms: χ^2^(1) = 0.3, *P* = 0.5721; 100–150 ms: χ^2^(1) = 1.1, *P* = 0.2871) and knee extensors (0–50 ms: χ^2^(1) = 3.6, *P* = 0.0593; 0–100 ms: χ^2^(1) = 1.0, *P* = 0.3082; 0–150 ms: χ^2^(1) = 0.6, *P* = 0.4491; 50–100 ms: χ^2^(1) = 0.01, *P* = 0.9446; 100–150 ms: χ^2^(1) = 0.1, *P* = 0.9136).

**Figure 3.**
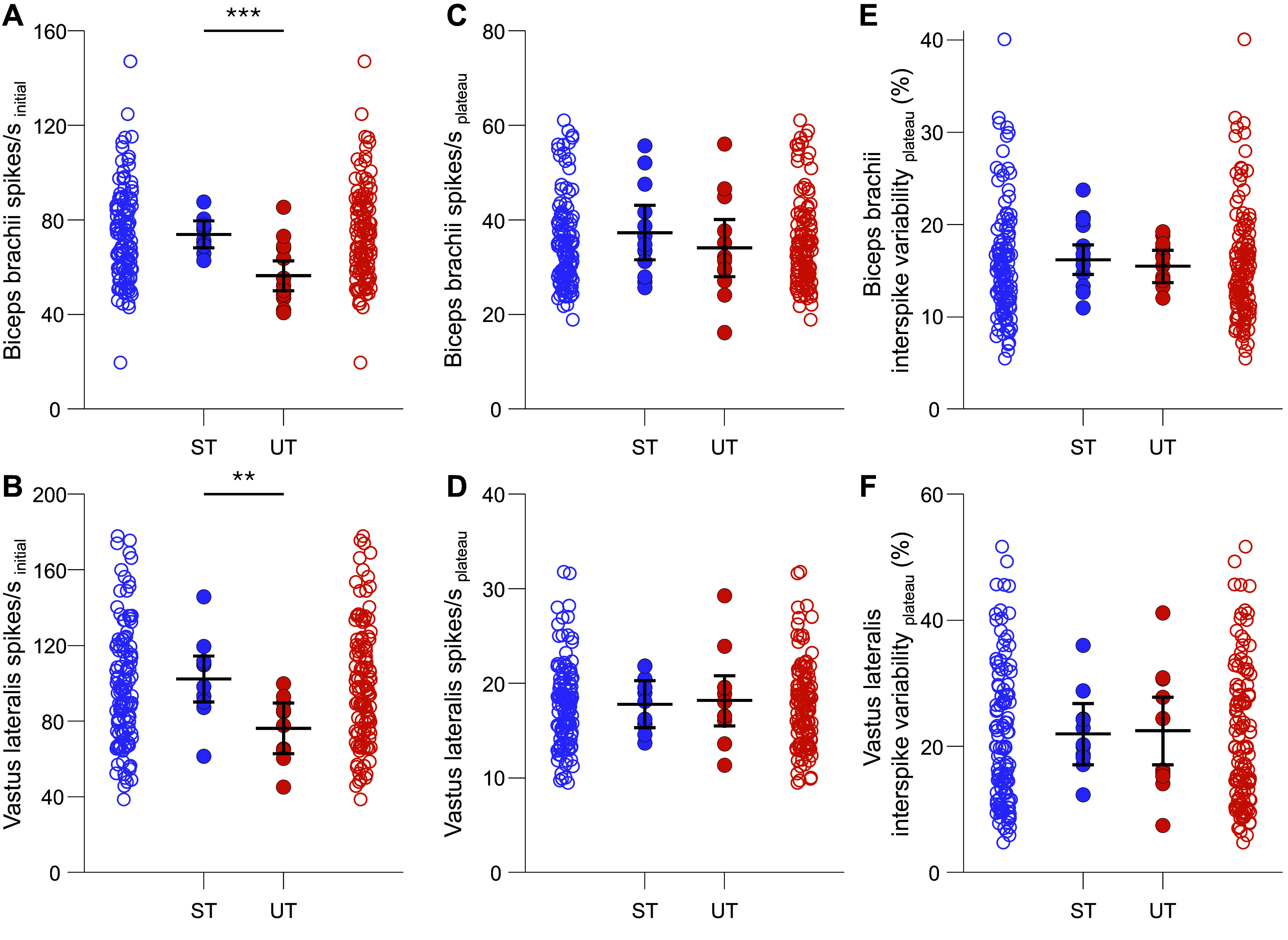
Motor unit discharge rate in biceps brachii and vastus lateralis during the initial (first three spikes; *A* and *B*) and the plateau phase (*C* and *D*), and the variability of the interspike interval (coefficient of variation, CV) during the plateau of rapid-hold contractions with the elbow flexors (*A*, *C*, and *E*) and knee extensors (*B*, *D*, and *F*), respectively, in strength-trained (ST; elbow flexion: *n* = 13, knee extension: *n* = 11) and untrained (UT; elbow flexion: *n* = 12, knee extension: *n* = 10) individuals. The lines with error bars represent the estimated marginal means with 95% confidence intervals, with ● denoting individual participant averages across three best contractions (based on force achieved in the first 100 ms), and the ○ denoting discharge rates of individual motor units within a group for the three best contractions. ***P < 0.001, **P < 0.01 relative to the other group.

**Figure 4.**
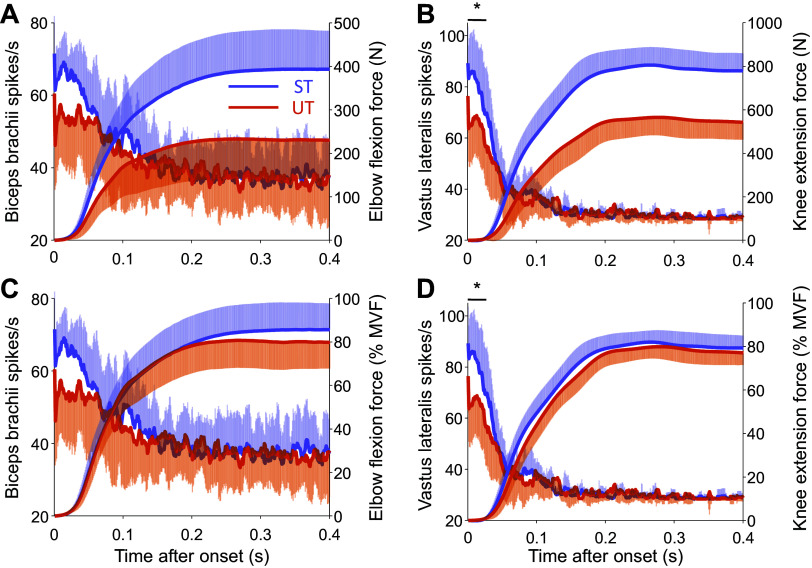
The average discharge rate per motor unit per second in biceps brachii and vastus lateralis along with force produced by elbow flexors and knee extensors. The average discharge rate per motor unit per second (with standard deviation) in a moving 35-ms window with a 1 sample overlap in biceps brachii (*A* and *C*) and vastus lateralis (*B* and *D*) during rapid-hold isometric elbow flexion (*A*—absolute, *C*—relative to maximal voluntary force, MVF) and knee extension (*B*—absolute, *D*—relative) contractions, respectively, in strength-trained (ST; elbow flexion: *n* = 13, knee extension: *n* = 11) and untrained (UT; elbow flexion: *n* = 12, knee extension: *n* = 10) individuals. Note that the *x*-axis denotes time after respective discharge rate (left *y*-axis) and force (right *y*-axis) onset. *P < 0.05 between-group difference in discharge rate.

The initial motor unit discharge rate, estimated over the first three interspike intervals following motor unit recruitment, was greater in strength-trained compared with untrained individuals for the biceps brachii (74 [68, 80] vs. 56 [50, 63] pulses per second (pps), *d* = 0.86, χ^2^(1) = 18.7, *P* < 0.0001; Fig. 3*A*, see Supplemental Table S2 for individual values; https://doi.org/10.6084/m9.figshare.26789488) and vastus lateralis (102 [90, 115] vs. 76 [63, 90] pps, *d* = 0.8, χ^2^(1) = 9.2, *P* = 0.0025; Fig. 3*B*, see Supplemental Table S2 for individual values; https://doi.org/10.6084/m9.figshare.26789488). The greater absolute force production of strength-trained individuals during rapid-hold contractions was also accompanied by a greater average number of discharges per motor unit per second (i.e., normalized cumulative discharge rate) in both biceps brachii (group × time interaction: χ^2^(1) = 33.2, *p* = 0.0002; Fig. 4, *A* and *C*) and vastus lateralis [group × time interaction: χ^2^(1) = 36.2, *P* < 0.0001; Fig. 4, *B* and *D*], with between-group differences for the 35 ms from the first firing evident in the vastus lateralis (77.0 [73.6, 80.4] vs. 62.8 [59.3, 66.4], *d* = 2.6, *P* < 0.0001), but not in the biceps brachii (65.7 [60.2, 71.3] vs. 53.6 [47.9, 59.4], *P* = 0.2716).

**Figure 5.**
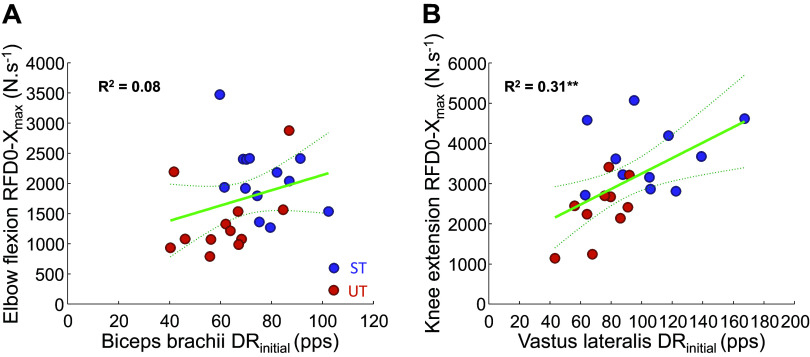
The association between maximal rate of force development of elbow flexors (*A*) and knee extensors (*B*) and the biceps brachii and vastus lateralis initial motor unit discharge rate during the performance of rapid-hold contractions in strength-trained (ST; elbow flexion: *n* = 13, knee extension: *n* = 11) and untrained (UT; elbow flexion: *n* = 12, knee extension: *n* = 10) individuals. The circles represent the mean score of discharge rate and maximal rate of force development across the three best contractions (based on force achieved in the first 100 ms). **P < 0.010 correlation.

In the context of the whole cohort, the initial discharge rate was associated with absolute maximal rate of force development for the knee extensors (*P* = 0.0094; Fig. 5), but not for the elbow flexors (*P* = 0.1707), with no association found for relative rate of force development in either muscle group (*P* ≥ 0.8305). There were no differences between groups in the discharge rate at the plateau of the contraction in either biceps brachii (37 [32, 43] vs. 34 [28, 40] pps, *d* = 0.76, χ^2^(1) = 0.7, *P* = 0.4177; Fig. 3*C*) or vastus lateralis (18 [15, 20] vs. 18 [16, 21] pps, *d* = 0.1, χ^2^(1) = 0.1, *P* = 0.8525; Fig. 3*D*). Similarly, no between-group differences were observed in the variability motor unit discharge timings (coefficient of variation of interspike interval) during the plateau phase of the rapid-hold contractions for both elbow flexors (16.2 [14.6, 17.8] vs. 15.5 [13.7, 17.2]%, *d* = 0.13, χ^2^(1) = 0.4, *P* = 0.5269; Fig. 3*E*) and knee extensors (22.0 [17.1, 26.8] vs. 22.5 [17.1, 27.8]%, *d* = 0.04, χ^2^(1) = 0.1, *P* = 0.8847; Fig. 3*F*).

## DISCUSSION

The aim of this cross-sectional study was to compare the rate of force development and motor unit discharge properties between cohorts with long-term history of strength training and untrained individuals. We observed a greater elbow flexor (*experiment 1*) and knee extensor (*experiment 2*) absolute rate of force development in rapid isometric contractions in the strength-trained cohorts compared with untrained controls. Despite greater initial motor unit discharge rate of rapid contractions of the long-term strength-trained individuals, this did not result in a greater relative rate of force development. These findings suggest that maximal motor neuron output during rapid contractions is greater for strength-trained compared with untrained individuals, but without a clear effect on the expression of the available force generating capacity (i.e., relative rate of force development).

Chronically strength-trained individuals had greater absolute elbow flexor and knee extensor rate of force development throughout the first 150 ms of the rapid voluntary contractions than untrained controls. Our results are consistent with previous cross-sectional studies, which observed greater absolute rate of force development in strength-trained individuals compared with controls (14, 15, 34). In particular, the magnitude of the difference in absolute rate of force development of chronically strength-trained versus untrained individuals, was aligned with that of previous studies which reported a similar pronounced difference in maximum strength (+64%, 34; +66%, 15) to that observed in the current study (+59% and +39%, in elbow flexion and knee extension, respectively). Conversely, the relative rate of force development (i.e., normalized to MVF) over the entire rising force-time curve did not differ between the two cohorts. Previous cross-sectional comparisons of chronically strength-trained individuals (>3–5 yr) showed contradictory results, reporting higher (14), or similar (15) early phase (first 50 ms), but lower late phase (≥75 ms; 15) relative rate of force development compared with untrained controls. In turn, discrepancies between these studies (14, 15) may be due to differences in maximum strength values of the involved trained cohorts (+23% in Ref. 14 vs. +66% in Ref. 15), with respect to the untrained counterparts, which may have influenced relative rate of force development expression. The magnitude of difference in maximum strength observed in the present study cohorts was greater than that of Orssatto et al. (14) for the knee extensors (+39%) and similar in the elbow flexors to those observed by Balshaw and coworkers (21; +66%). Therefore, our findings indicate a similar ability to rapidly utilize the available force generating capacity between long-term strength-trained individuals and controls.

The relative rate of force development did not differ between the strength-trained and untrained group, despite the greater motor unit discharge rate of strength-trained individuals. There are multiple possible explanations for the greater neural drive without parallel differences in the relative rate of force development of trained participants. Namely, despite motor unit discharge rate having been shown to play a role in the rate of force development (12, 13), its importance may be considered secondary to other mechanisms limiting the rate of force development. Though initial experimental studies suggested a strong relationship between the rate of force development and MU discharge rate (13), subsequent simulations (35) indicated that the time span of motor unit recruitment, rather than discharge rate, may play a significantly greater role in determining the rate of force development, especially in the early phase of the rising force-time curve. Indeed, we only showed a modest correlation between motor unit discharge rate and rate of force development for the knee extensors, but not elbow flexors. These results suggest that differences in motor unit discharge rate are not sufficient to explain the performance of rapid contractions. Alternatively, it is important to note that our investigation focused on the MU behavior of a single muscle without regard for the potential differences in the MU behavior of synergist and/or antagonist muscles that could have contributed to the lack of differences between the groups. This might explain the comparatively smaller correlation than shown previously in the tibialis anterior (13), a muscle with a comparatively smaller contribution of synergist muscles to joint torque (36) than muscle groups investigated in the presented study.

Furthermore, the lack of between-group differences in the relative rate of force development despite greater neural drive of strength-trained individuals could also be due to the opposing effect of multiple years of maximum strength training on the intrinsic contractile muscle properties. We have previously found a lower relative rapid force production and increased time-to-peak-tension of evoked contractions (i.e., a slowing of the intrinsic contractile properties) after 12 wk of strength training (37) and for chronically trained versus untrained (15). In particular, the slowing of intrinsic contractile properties is likely the result of a decreased myosin heavy-chain type IIX expression induced by prolonged maximum strength training exposure (38, 39), which is in turn associated with slower evoked contractile properties and rate of force development (40, 41). The present results suggest that the nervous system of chronically strength-trained individuals may partly compensate for the training-induced overall slowing of contractile muscle properties by augmenting the discharge rate and neural drive directed to the muscles in the very early phase (<50 ms) of contraction leading to similar (relative) explosive strength compared with controls. However, it is possible that the higher discharge capacity of motoneurons of chronically strength-trained individuals could be due to other neural changes induced by training, e.g., afferent (42) and supraspinal inputs to motor neurons (43), which could arise from different cortical and subcortical pathways, with the precise mechanisms that led to the greater motoneuron output after long-term strength training remaining to be elucidated. Though speculative, another possibility is that during the performance of MVF the activation patterns differ to that during rapid contraction, with sufficient time for greater contribution of efferent and, in particular, afferent synaptic inputs, yielding disproportionally greater MVF relative to rate of force development in strength-trained individuals.

We observed that two separate cohorts of long-term strength-trained participants had a greater initial motor unit discharge rate (i.e., average of the first 3 interspike intervals) in both biceps brachii and vastus lateralis muscles, and a greater normalized cumulative discharge rate in the initial period of the contraction compared with untrained controls. However, these between-group differences were not observed when considering the plateau phase of the contraction. It is not entirely clear why differences between the groups are only apparent in the initial period of motor unit discharge rate. Though speculative, strength-trained individuals have been shown to exhibit enhanced indices of reticulospinal function (7, 43), with the reticular formation having been implicated in the rapid transmission of excitatory input and augmented motoneuron output during rapid contractions (20), which might contribute to the greater, but short-lived, initial motor unit discharge rate where the contribution of afferent input is likely to be limited (13). Conversely, when considering the plateau phase of the contraction at ∼80% MVF, the results were similar to our previous comparisons of strength-trained and untrained individuals during submaximal and maximal isometric contractions (10, 11). The differences in absolute forces between the groups, despite the lack of difference in motor unit discharge rate, are likely explained by the superior muscle morphology of strength-trained individuals (4). In this respect, as previously reported (10), the strength-trained participants involved in the present study (*experiment 1*) had a ∼72% greater biceps brachii maximum anatomical cross-sectional area compared with controls.

The current study provides novel insights about how motor unit discharge rate differs in individuals exposed to long-term strength training. Nevertheless, there are some limitations that should be recognized. First, the cross-sectional nature of the present study inevitably provides a weaker level of evidence than that of a longitudinal design. Consequently, the relative contribution of innate characteristics of the volunteers cannot be separated from the influence of prolonged strength training on the observed motor unit characteristics during explosive contractions. Second, in this study we focused primarily on the motor unit discharge properties and functional differences. A more comprehensive study including additional variables, such as intrinsic muscle contractile properties and muscle size may be required in the future. Third, it should be noted that the participants of both strength-trained cohorts were recruited based on their >3 yr of strength-training experience. Therefore, some within-group variability may have been introduced by the fact that the volunteers did not complete a uniform (i.e., standardized) strength training program, but performed variable individualized programs including different combinations of both slow, heavy and explosive strength training among many other training variables (see *Participants*). Finally, although we adopted the most up-to-date approach (13) to record and study the activity of populations of motor units during rapid contractions, it has to be noted that relatively few motor units were identified per subject in both muscles. This precluded a reliable assessment of the speed of motor unit recruitment, which has been shown to be one of the key determinants of rate of force development (35).

In conclusion, we assessed the rate of force development and motor unit discharge characteristics during rapid contractions in chronically strength trained individuals who exhibited a greater absolute rate of force development, a higher initial discharge rate and a greater average number of motor unit discharges per second in the early phase of rapid contractions. Interestingly, despite the augmented output of the spinal cord, this did not result in a greater relative rate of force development, likely due to the opposing effect of prolonged strength training on slowing of the intrinsic contractile muscle properties. Overall, the present study provides evidence for a higher maximal motor unit discharge rate in chronically strength-trained individuals.

## DATA AVAILABILITY

Data will be made available upon reasonable request.

## SUPPLEMENTAL MATERIAL

10.6084/m9.figshare.26789488 Supplemental Tables S1 and S2: https://doi.org/10.6084/m9.figshare.26789488.

## GRANTS

J.Š. is supported by Versus Arthritis Foundation Fellowship under Reference No. 22569. S.M. is supported by the Japan Society for the Promotion of Science under Grant No. 18K17837. A.H. is supported by Slovenian Research Agency under Grant Nos. J2-1731 and P2-0041, and Horizon Europe Research and Innovation Programme under Grant No. 101079392.

## DISCLOSURES

No conflicts of interest, financial or otherwise, are declared by the authors.

## AUTHOR CONTRIBUTIONS

J.Š., A.C., A.H., D.F., J.P.F., and A.D.V. conceived and designed research; J.Š., A.C., T.G.B., S.M., M.B.L., and A.D.V. performed experiments; J.Š., A.C., and A.H. analyzed data; J.Š., A.C., D.F., J.P.F., and A.D.V. interpreted results of experiments; J.Š. prepared figures; J.Š. and A.C. drafted manuscript; J.Š., A.C., T.G.B., S.M., M.B.L., A.H., D.F., J.P.F., and A.D.V. edited and revised manuscript; J.Š., A.C., T.G.B., S.M., M.B.L., A.H., D.F., J.P.F., and A.D.V. approved final version of manuscript.
